# Monocyte and Macrophage Lipid Accumulation Results in Down-Regulated Type-I Interferon Responses

**DOI:** 10.3389/fcvm.2022.829877

**Published:** 2022-02-10

**Authors:** Lisa Willemsen, Hung-Jen Chen, Cindy P. A. A. van Roomen, Guillermo R. Griffith, Ricky Siebeler, Annette E. Neele, Jeffrey Kroon, Marten A. Hoeksema, Menno P. J. de Winther

**Affiliations:** ^1^Department of Medical Biochemistry, Experimental Vascular Biology, Amsterdam Cardiovascular Sciences, Amsterdam Infection and Immunity, University of Amsterdam, Amsterdam, Netherlands; ^2^Department of Experimental Vascular Medicine, Amsterdam Cardiovascular Sciences, University of Amsterdam, Amsterdam, Netherlands

**Keywords:** atherosclerosis, macrophage, monocyte, foam cell formation, cholesterol, inflammation, interferon response, immunometabolism

## Abstract

Macrophages are critical components of atherosclerotic lesions and their pro- and anti-inflammatory responses influence atherogenesis. Type-I interferons (IFNs) are cytokines that play an essential role in antiviral responses and inflammatory activation and have been shown to promote atherosclerosis. Although the impact of type-I IFNs on macrophage foam cell formation is well-documented, the effect of lipid accumulation in monocytes and macrophages on type-I IFN responses remains unknown. Here we examined IFN stimulated (ISG) and non-ISG inflammatory gene expression in mouse and human macrophages that were loaded with acetylated LDL (acLDL), as a model for foam cell formation. We found that acLDL loading in mouse and human macrophages specifically suppressed expression of ISGs and IFN-β secretion, but not other pro-inflammatory genes. The down regulation of ISGs could be rescued by exogenous IFN-β supplementation. Activation of the cholesterol-sensing nuclear liver X receptor (LXR) recapitulated the cholesterol-initiated type-I IFN suppression. Additional analyses of murine *in vitro* and *in vivo* generated foam cells confirmed the suppressed IFN signaling pathways and suggest that this phenotype is mediated *via* down regulation of interferon regulatory factor binding at gene promoters. Finally, RNA-seq analysis of monocytes of familial hypercholesterolemia (FH) patients also showed type-I IFN suppression which was restored by lipid-lowering therapy and not present in monocytes of healthy donors. Taken together, we define type-I IFN suppression as an athero-protective characteristic of foamy macrophages. These data provide new insights into the mechanisms that control inflammatory responses in hyperlipidaemic settings and can support future therapeutic approaches focusing on reprogramming of macrophages to reduce atherosclerotic plaque progression and improve stability.

## Introduction

Cardiovascular disease remains the leading cause of death globally with atherosclerosis as the major underlying cause (1, 2). Atherosclerosis is initiated by endothelial dysfunction caused by conventional risk factors such as hypercholesterolemia, high blood pressure, smoking, a lack of exercise, and an unhealthy diet (3–7). Familial hypercholesterolemia (FH) patients have elevated levels of serum low density lipoprotein (LDL) that have been associated with premature atherosclerosis (1, 3–5). Lifelong LDL cholesterol-lowering treatment effectively reduces cardiovascular events in FH patients.

In atherogenesis, LDL is modified within the arterial wall triggering endothelial and immune cell activation and subsequent recruitment of immune cells like monocytes (8). When monocytes enter the arterial intima, they differentiate into macrophages (9). The complex atherosclerotic microenvironment drives the formation of multiple macrophage subsets, including inflammatory and foamy macrophages (10–14). The various functions that macrophages can acquire are essential for atherosclerotic plaque development, stability and clinical outcome (9, 15, 16).

Macrophages can take up excessive amounts of modified LDL [e.g., oxidized (oxLDL) and acetylated LDL (acLDL)] using scavenging receptors causing macrophage foam cell formation (17). Lipid accumulation in foamy macrophages activate nuclear receptors, including the liver X receptor (LXR) (18). LXRs stimulate lipid efflux *via* upregulation of the lipid ATP-binding cassette transporters ABCA1 and ABCG1, but are also important for macrophage survival and immune responses (19, 20). LXR activation antagonizes NF-κB signaling and its deficiency decreases control of intracellular bacterial growth in macrophages (21).

Under homeostatic conditions, immune cells maintain low-levels of IFN-β in an autocrine fashion that is required for a rapid response to environmental cues, e.g., the production of other type-I IFNs (IFN-α/β) and its downstream signaling pathways (22). Therefore, in response to intra-and extracellular stimulation of pattern recognition receptors (PRR) with foreign substances, immune cells are capable of producing large amounts of type-I IFNs. Once secreted, type-I IFNs bind to their receptors (IFNAR1/2) on nearby cells and thereby leading to the phosphorylation and nuclear translocation of transcription factors such as Signal Transducer and Activator of Transcription (STATs) and IFN regulatory factors (IRFs) (23, 24). IRFs and STATs can form complexes and bind to DNA sequences containing IFN-sensitive response element (ISRE) triggering a diverse group of IFN-stimulated genes (ISGs) with various functions (25). Of note, IRF7 is itself an ISG, but also can bind to the promoter region of *IFNB1* and *IFNA* and thereby serves as one of the key regulators of type-I IFN autocrine feedback loop (26–28).

Studies have investigated the effect of IFN-α/β and its downstream signaling on lipid metabolism in monocytes and macrophages. While evidence suggested that IFN stimulation reduced cholesterol synthesis in macrophages (29), many studies showed type-I IFN exposure triggered cholesterol uptake (30, 31), lipid accumulation (32) and foam cell formation (30, 33). Furthermore, in a mouse model for atherosclerosis, IFN-β treatment accelerated lesion formation whereas myeloid-specific IFNAR1 deletion resulted in a more favorable atherosclerotic phenotype (34), suggesting a pro-atherogenic feature of type-I IFNs. However, the role of lipid exposure and metabolism on the type-I IFN response is still unknown. By defining this mechanistic link, macrophage subsets may be amended toward desired phenotypes using clinical therapeutic agents. In this way, reprogramming of macrophages can be applied to reduce atherosclerotic plaque progression and improve stability.

In this study, we demonstrate that lipid-loaded foamy macrophages of mice and men show perturbated type-I IFN responses caused by defective IFN-β production. This suppressed IFN response can be rescued by exogenous IFN-β treatment. Furthermore, we demonstrate that monocytes of untreated FH patients also show a deactivated IFN signature. In these FH patients, lipid-lowering treatment restored the type-I IFN response. These findings are of considerable interest for the understanding of regulation of macrophages in the context of lipid-related diseases, like atherosclerosis and FH, and viral infections.

## Materials and Methods

### Mice

*Ldlr*^−/−^ mice (on a C57Bl/6 background) were housed at the Animal Research Institute Amsterdam UMC (ARIA). All mice experiments were conducted after approval of the Committee for Animal Welfare (University of Amsterdam).

### Bone Marrow-Derived Macrophages

Bone marrow cells were isolated from the hind limbs of C57Bl/6 mice and cultured in RPMI-1640 medium, with 10% heat inactivated fetal bovine serum (FBS), penicillin (100 U/ml), streptomycin (100 μg/ml), 2 mML-glutamine (all purchased from ThermoFisher), and 15% L929-conditioned medium containing M-CSF. Bone marrow-derived macrophages (BMDMs) were generated by culturing the cells for 7 days. Next, BMDMs were loaded overnight with 50 μg/mL human acetylated LDL (KyvoBio) to induce macrophage foam cell formation, and were the next day stimulated with 10 ng/mL lipopolysaccharide (LPS from *Escherichia coli*; O111:B4; Sigma) or 50 ng/mL rmIFN-β (R&D Systems 8234-MB) as indicated (for 6 or 24 h). For serial dilution experiment, rmIFN-β was applied with the concentration as indicated in the figure. When indicated, BMDMs were stimulated with 2 μM LXR-agonist GW3965 (Sigma) for 17 h.

### IFN-β ELISA

Non-foamy and acLDL-loaded BMDMs prepared as described above, followed by LPS stimulation for 6 h. Supernatant were collected and the IFN-β concentration was measured using the mouse IFN-beta DuoSet enzyme-linked immunosorbent assay (ELISA) kit (R&D Systems) according to manufacturer's protocol with no additional dilution.

### Human Monocyte-Derived Macrophages

Buffy coats of healthy anonymous blood donors were obtained from Sanquin blood bank in Amsterdam, the Netherlands. All the subjects provided written informed consent. Human monocyte-derived macrophages (hMDMs) were prepared as previously described (35). In short, CD14^+^ monocytes were isolated with Lymphoprep™ (Axis-Shield) followed by MACS CD14 magnetic beads (Miltenyi) purification. The resulting monocytes were seeded at a density of 0.8 million cells/well on 24-well tissue culture plates (Greiner) and differentiated to macrophages with 50 ng/mL human M-CSF (Miltenyi) for 6 days in Iscove's Modified Dulbecco's Medium (life technology) containing 10% heat-inactivated fetal bovine serum (Gibco), 1% penicillin/streptomycin solution (Gibco) and 1% L-glutamine solution (Gibco). After differentiation, hMDMs were loaded 18 h with 50 μg/mL human acetylated LDL (Invitrogen) followed by 50 ng/mL IFN-β (R&D) stimulation or remained untreated.

### Gene Expression Analysis by qPCR

Total RNA was isolated using the GeneJET RNA Purification kit (Thermo). cDNA synthesis was then performed using the iScript cDNA synthesis kit (Biorad), followed by quantitative real-time PCR with Sybr Green Fast Mix. qPCR was performed on a Viia7 Real-time PCR system (Applied Biosystems). The delta-delta Ct (2^−ΔΔCt^) method was used to calculate the relative fold change of qPCR data using the housekeeping genes: *HPRT1* and *RACK1* for human, and *Actb, Gapdh*, and *Ptgs1* for mouse data. Primer sequences are shown in Supplementary Table 3.

### RNA Sequencing and Bioinformatics for BMDMs

RNA was isolated from BMDMs using the RNeasy Mini Kit (QIAGEN) with DNase treatment. 700 ng RNA was used for Illumina library construction. RNA amplification, cDNA generation, and adaptor ligation were performed using the KAPA mRNA HyperPrep Kit (Roche) following the manufacturer's instructions. Samples were pooled, diluted to 10 nM, and sequenced single-end on an Illumina HiSeq 4,000 system (Illumina) to a depth of ± 20 million reads with a length of 50 base pairs. Reads were aligned to the mouse genome mm10 by STAR 2.5.2b with default settings (36). BAM files were indexed and filtered on MAPQ >15 with v1.3.1 SAMtools (37). Raw tag counts and reads per kilobase million (RPKM) per gene were summed using HOMER2's analyzeRepeats.pl script with default settings and the -noadj or -rpkm options for raw counts and RPKM reporting (38). Differential expression was assessed using the DESeq2 Bioconductor package in an R 3.6.3 environment (39).

### Familial Hypercholesterolemic Patients and Healthy Subjects

The study population, design, and further processing of these human study subjects and their samples have been extensively described (40). Briefly, untreated FH patients who indicated to start lipid-lowering therapy (statin, PCSK9 antibody, and/or ezetimibe) according to their treating physician were included. The healthy controls were age, sex, and body mass index (BMI) matched with the FH patients. After inclusion, FH patients fasted for at least 9 h before blood samples were drawn for lipid measurements and monocyte isolation. This was repeated after 12 weeks of lipid-lowering therapy. The healthy controls underwent these procedures once. All participants provided written informed consent. The study protocol was approved by the ethics committee of the Amsterdam UMC and was conducted according to the principles of the Declaration of Helsinki.

### RNA Sequencing and Bioinformatics for Human Monocytes

Monocytes were isolated as described above. Monocytes were lysed using TriPure (Sigma Aldrich) and stored at −80°C until further processing. For RNA isolation, 0.2 mL chloroform was added per mL of TriPure. Next, samples were spinned at 12,000 g for 15' at 4°C. Subsequently, the aqueous phase was added to 450 μl isopropanol containing GlycoBlue. Next, tubes were shaken vigorously, chilled for 30 min at −20°C and centrifuged at 12,000 g for 10' at 4°C. RNA pellets were washed twice with 75% ethanol and pellets were air-dried at RT and resuspended in nuclease-free H_2_O. RNA-seq libraries were prepared, including rRNA depletion, by using the NEBNext Ultra II Directional RNA Library Prep Kit for Illumina according to manufacturer's instructions. Poly-A containing transcripts were sequenced on an Illumina Novaseq 6,000 instrument to a depth of ± 20 million reads by GenomeScan. Reads were aligned to the human reference genome (hg38) using a short-read aligner based on Burrows-Wheeler Transform with default settings (41). Binary alignment map (BAM) files were sorted on coordinates and indexed with the samtools v1.3 package (37). Normalized read count values were calculated. Differential expression was assessed using the DESeq2 Bioconductor package in an R V.3.6.3 programming environment with gene expression called differential with a false discovery rate (FDR) <0.05 and a median read count >1 in at least one group (39). Presented normalized counts were tested using one-way analysis of variance (ANOVA) followed by Bonferroni's comparisons test.

### Genome-Wide Transcriptomic Data Analysis

Upstream regulator analysis and regulatory network analysis were performed on Ingenuity Pathway Analysis (Ingenuity System Inc., USA). Pathway overrepresentation analysis was conducted on Meta scape platform [http://metascape.org; (42)]. Known transcription factor motif analysis on gene subsets was performed by using HOMER (v4.11) with the following setting: findMotis.pl “genelist” -start−200 -end 100 -len 8, 10, 12 (38).

### Data Availability

Public transcriptomic data sets used in the current study are available in the Gene Expression Omnibus (GEO): (1) GSE118656: acLDL-loaded BMDMs (43) (2) GSE42061: peritoneal macrophages derived from wildtype or *Apoe*^−/−^ mouse (44), and (3) GSE6054: Monocytes from familial hypercholesterolemia patients (45). RNA-seq data of the BMDMs treated with the LXR-agonist GW3965 or DMSO are deposited in the Gene Expression Omnibus (GEO) under the accession number: GSE193118. RNA-seq data of the monocytes from familial hypercholesterolemia patients and healthy subjects are deposited in GEO under the accession number: GSE192709 (processed data) and EGA (raw data).

### Statistical Analysis

Except genome-wide transcriptomic data, statistical analyses were performed using GraphPad Prism 9.1.0 (GraphPad Software). For single comparison tests, paired or unpaired *t*-tests were applied based on the experiment design. For multiple comparison tests, one-way, two-way analysis of variance (ANOVA) or multiple *t*-tests were conducted on the basis of the addressed question.

## Results

### Macrophage Foam Cell Formation Leads to Decreased Expression of IFN-β and Its Targets

**Figure 1:**
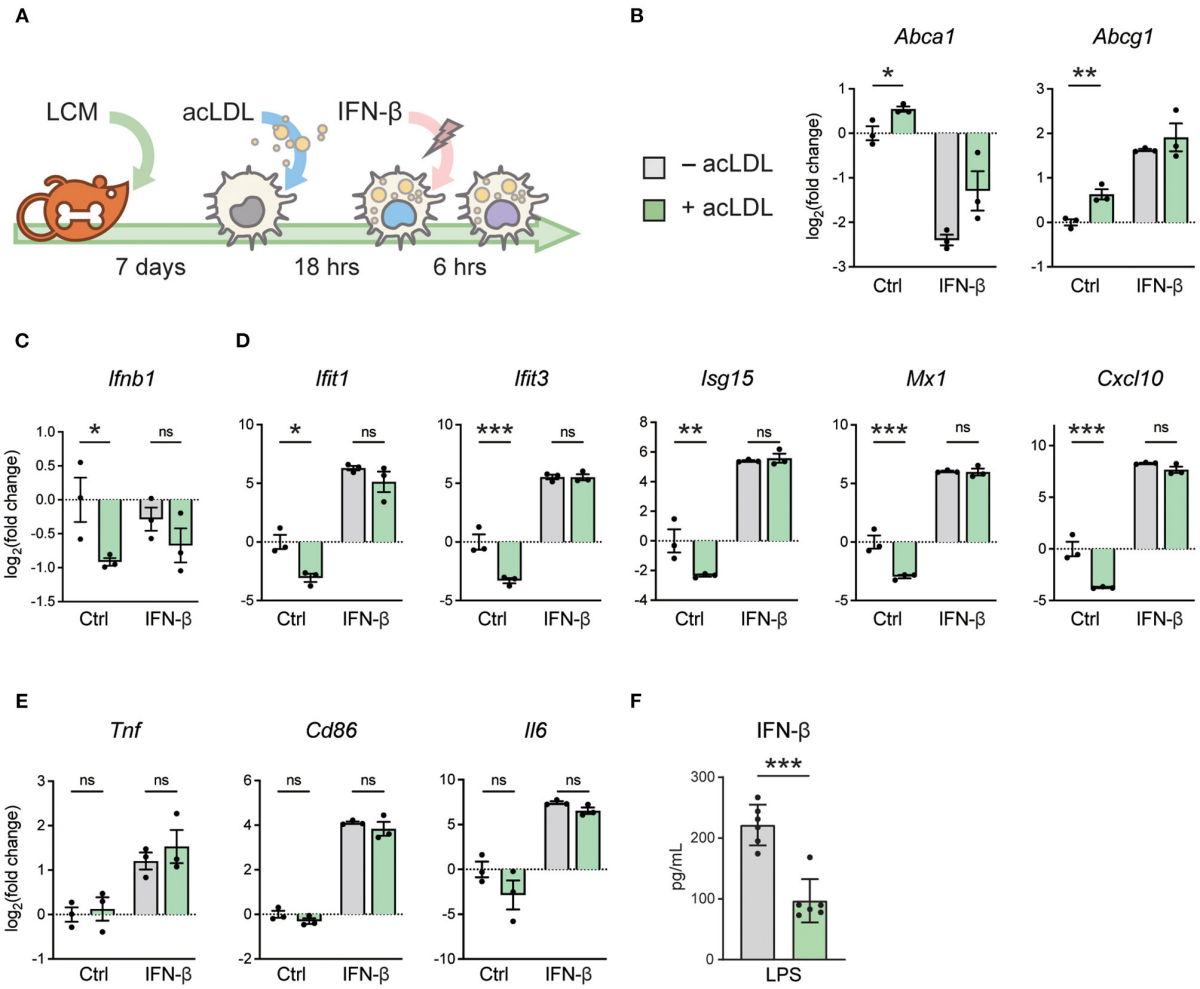
acLDL exposure suppresses type-I IFN gene programs in BMDMs. **(A)** Schematic plot showing the protocol of generating foamy BMDMs with 16-h (50 μg/mL) acLDL exposure. Subsequently, these foamy macrophages were treated with (50 ng/mL) or without IFN-β for 6 h and mRNA expression of type-I interferon genes was determined. **(B)** mRNA expression of the cholesterol efflux transporters *Abca1* and *Abcg1* was measured as a control for lipid loading using qPCR. **(C)** *Ifnb1* mRNA expression of unstimulated, acLDL, and/or IFN-β stimulated BMDMs measured by qPCR. **(D)** *Ifit1, Ifit3, Isg15, Mx1*, and *Cxcl10* mRNA expression of unstimulated, acLDL and/or IFN-β stimulated BMDMs measured by qPCR. **(E)** *Tnf*, *Cd86*, and *Il6* mRNA expression of unstimulated, acLDL and/or IFN-β stimulated BMDMs. **(F)** IFN-β production in supernatant of foamy and non-foamy BMDMs that were stimulated with LPS for 6 h. ns, not significant, ^*^P < 0.05, ^**^P < 0.01, ^***^P < 0.001. **(B–E)** *n* = 3 and **(F)** *n* = 6 biological replicates per group.

To determine the effect of macrophage foam cell formation on type-I IFN responses, murine bone marrow cells were differentiated to macrophages (BMDMs) and subsequently treated with acLDL or left untreated as control. Foamy and non-foamy macrophages were subsequently stimulated with IFN-β or kept untreated for 6 h (Figure 1A). Macrophage foam cell formation resulted in a significant upregulation of the cholesterol efflux transporter genes *Abca1* and *Abcg1*, compared to non-foamy macrophages, indicating proper foam cell formation (46); (Figure 1B). Interestingly, we found that macrophage lipid loading significantly suppressed the transcription of *Ifnb1* (Figure 1C), as well as several members of its downstream ISGs, including IFN-induced protein with tetratricopeptide repeats 1 (*Ifit1*), *Ifit3, Isg15*, MX dynamin like GTPase 1 (*Mx1*), C-X-C motif chemokine ligand 10 (*Cxcl10*), *Ccl5* and *Cxcl9* (Figure 1D; Supplementary Figure 1A). Remarkably, most of these differences disappeared after subsequent stimulation with exogenous IFN-β suggesting that foam cells remained responsive to IFN-β, while some differences persisted (Supplementary Figure 1A). This cholesterol loading-induced immunomodulation seemed to be IFN-specific since other pro-inflammatory genes, such as *Tnf*, *Cd86*, and *Il6* were not affected (Figure 1E). Furthermore, IFN-responsive transcription factors, *Stat1, Stat2*, and *Irf7* show the same regulation pattern as the ISGs (Supplementary Figure 1B). To test whether IFN-β secretion was down regulated by foam cell formation, we stimulated macrophages with LPS and found IFN-β secretion to be significantly decreased after acLDL-loading compared to controls (Figure 1F). This indicates that macrophage foam cell formation hampers the endogenous IFN pathways.

### Exogenous IFN-β Treatment Rescues the Cholesterol-Initiated Type-I IFN Suppression

**Figure 2:**
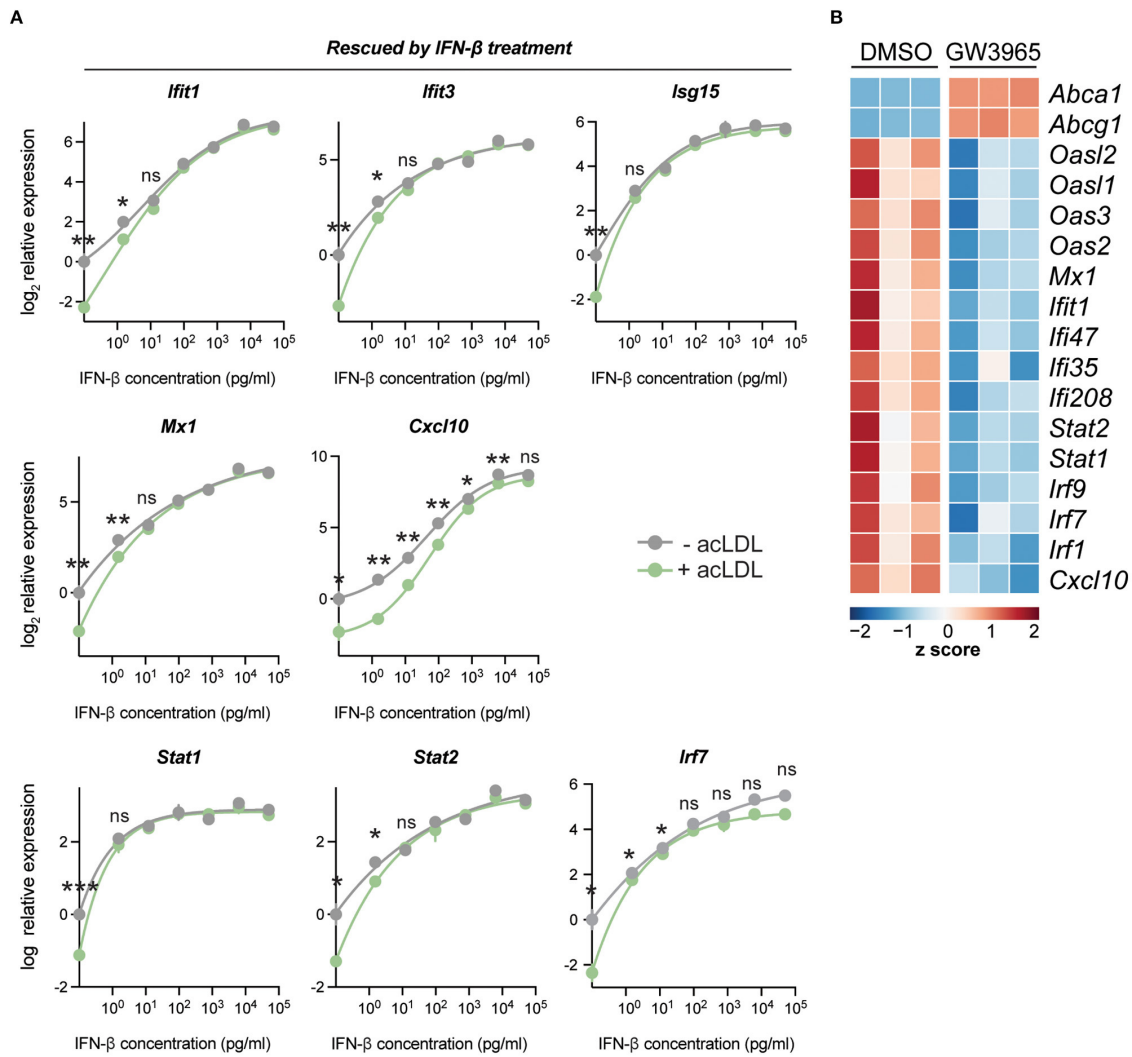
IFN-β exposure reverses acLDL-suppressed type-I interferon response in BMDMs. **(A)** mRNA expression of type-I interferon stimulated genes in BMDMs after a total of 24 h of acLDL exposure combined with different concentrations (1.5 pg/mL to 50 ng/mL) of IFN-β for 6 h of stimulation. The transcriptional suppression of *Cxcl10, Isg15, Ifit1, Ifit3, Stat1, Stat2*, and *Mx1* that was induced by acLDL loading was reversed after IFN-β exposure. **(B)** Heatmap indicating the row z score of the cholesterol loading-responsive genes *Abca1* and *Abcg1*, and genes the type-I interferon response of BMDMs treated with LXR agonist (GW3965) or DMSO for 17 h. **(A,B)** *n* = 3 biological replicates per group. ns, not significant, ^*^P < 0.05, ^**^P < 0.01.

To determine whether the suppression of ISGs was solely caused by the reduced IFN-β production in the context of lipid loading, we tested whether the expression of the ISGs changed when different doses of exogenous IFN-β were applied on foam cells and control macrophages. A concentration range (from 1.5 pg/mL to 50 ng/mL) of IFN-β was administered to acLDL-loaded and untreated mouse BMDMs. In line with our previous observations, acLDL loading increased the expression of *Abca1* and *Abcg1* (Supplementary Figure 2A), and many ISGs including *Ifit1, Ifit3, Isg15, Mx1, Cxcl10, Stat1, Stat2*, and *Irf7* were suppressed by acLDL loading which suppression was rescued by exogenous IFN-β treatment (Figure 2A). Moreover, a strong dose-dependent effect of IFN-β on the ISGs was observed, although a few ISGs (*Cxcl9* and *Ccl5*) were not rescued by IFN-β administration (Supplementary Figure 2B) whereas the non-ISG pro-inflammatory cytokine *Il6* again showed no differences with or without acLDL loading (Supplementary Figure 2C). Previous studies have shown that macrophages maintain constitutive production of low levels of type-I IFNs for rapid response to pathogen activation (22). Our data suggest that lipid-loading disrupts this basal macrophage type-I IFN autocrine/paracrine loop through suppressing the homeostatic production of IFNs.

### Stimulation of the Cholesterol-Sensing Nuclear Receptor LXR Recapitulates the Cholesterol-Initiated Type-I IFN Suppression

Liver X receptors (LXRs) are cholesterol-sensing transcription factors regulating lipid metabolism and transport, also impacting on inflammatory signaling in macrophages (19). LXR activation is a classical transcriptional response upon lipid loading (18). To determine whether the cholesterol-initiated type-I IFN suppression might be mediated *via* LXR, a synthetic LXR agonist (GW3965) was administered to BMDMs. Interestingly, LXR activation led to a clear suppression of ISGs, a signature that resembles that of lipid-laden macrophages (Figure 2B). This indicates that the lipid-driven type-I IFN suppression may be mediated through LXR activation.

### Lipid-Loading Affects the Expression of ISGs Associated With IRF Promoter Motifs

**Figure 3:**
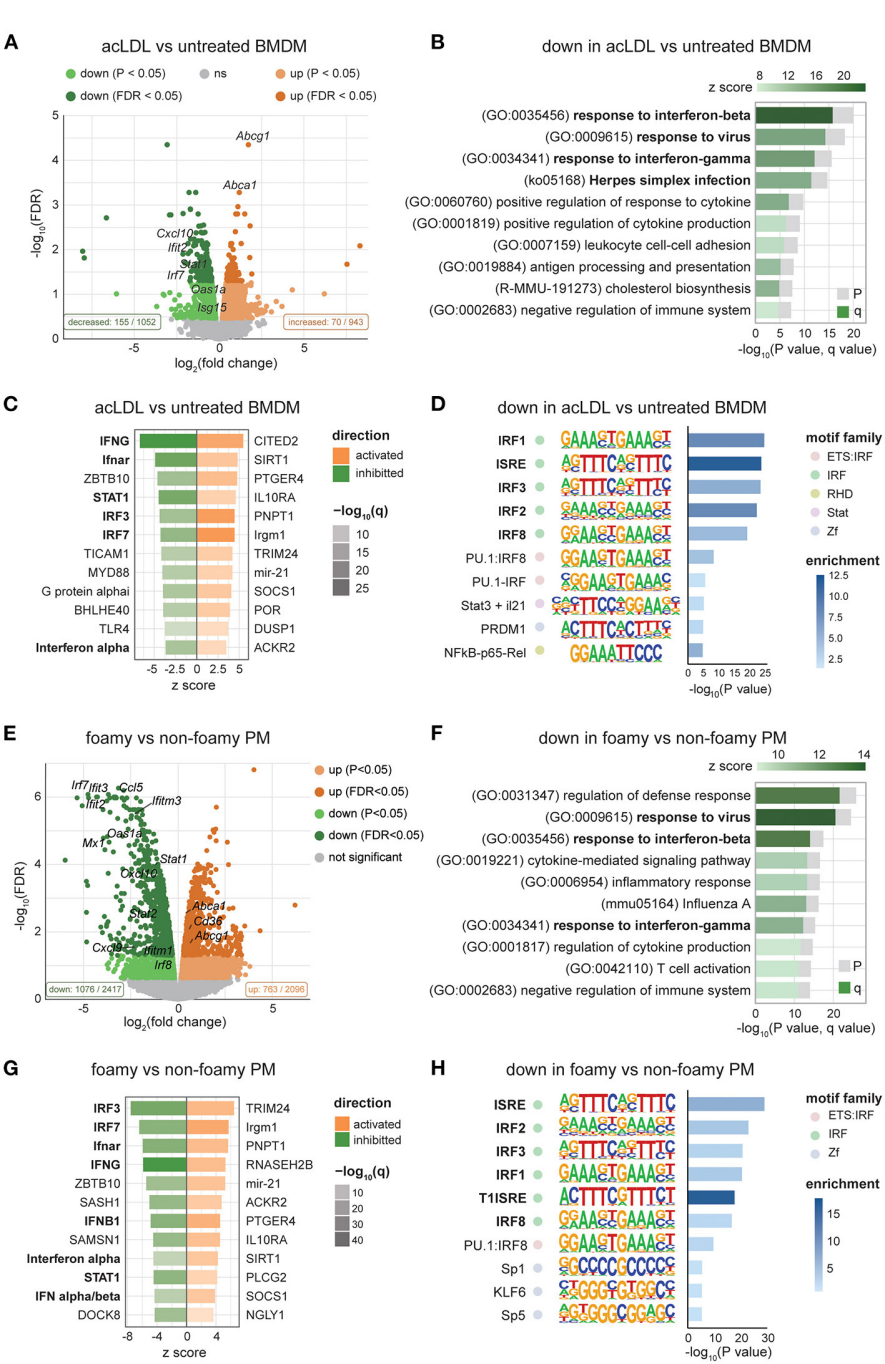
Transcriptomic analysis shows suppressed IFN signaling in foamy macrophages in different mouse models. **(A)** Volcano plot of RNA-seq data showing the log_2_FC and -log_10_(FDR) of acLDL-treated macrophages with downregulated genes in green and upregulated genes in orange. **(B)** Pathway enrichment analysis of significantly downregulated genes (FDR < 0.05) in acLDL-loaded macrophages. **(C)** Upstream regulators predicted by the Ingenuity Pathway Analysis (IPA) software of acLDL-loaded vs. untreated macrophages. **(D)** Motif enrichment analysis showed an enrichment of interferon-related motifs among the down-regulated genes in acLDL-loaded macrophages. **(E)** Volcano plot of RNA-seq data showing the log_2_FC and -log_10_ (FDR) of peritoneal macrophages (PMs) derived from *Apoe*^−/−^ compared to WT, with downregulated genes in green and upregulated genes in orange. **(F)** Pathway enrichment analysis of significantly downregulated genes (FDR < 0.05) in *Apoe*^−/−^ PMs. **(G)** Upstream regulators predicted by the IPA software of *Apoe*^−/−^ vs. WT PMs. **(H)** Motif enrichment analysis showed an enrichment of interferon-related motifs among the down-regulated genes in *Apoe*^−/−^ PMs. **(A–D)** raw data obtained from GSE118656 and **(E–H)** GSE42061.

To further explore the underlying mechanism of the lipid-induced IFN suppression, we analyzed the transcriptome of acLDL-treated and untreated BMDMs using a publicly available dataset (GSE118656) (43). In line with our data, we found decreased ISG expression (*Ifit2, Isg15, Cxcl10, Oas1a, Irf7*, and *Stat1*) in acLDL-loaded macrophages (Figure 3A). Pathway analysis of significantly down regulated genes showed that the responses to IFNs and viral infections were the most affected biological processes (Figure 3B), while lipid metabolism was a top hit in the upregulated genes (Supplementary Figure 3A). Furthermore, upstream regulator analysis identified IFNs (IFN-α and IFN-γ), type-I IFN receptor (Ifnar), and the transcription factors STAT1, IRF3, and IRF7 as the most inhibited upstream regulators in acLDL-loaded macrophages (Figure 3C, green bars). IRF3 and IRF7 are the key transcription factors that mediate the transcription of type-I IFNs (15, 47, 48). The importance of IRFs in the lipid-driven type-I IFN suppression was confirmed by a constructed regulatory network of the foamy BMDMs transcriptome (Supplementary Figure 3C). Moreover, the anti-inflammatory macrophage-associated upstream regulators SIRT1 (49, 50), SOCS1 (51), and IL-10 receptor (IL10R) (52–54) were activated in the foamy BMDMs (Figure 3C, orange bars). Furthermore, studies have shown that these regulatory factors suppress IFN responses (49, 51, 52, 54), confirming the suppressive role of cholesterol accumulation to type-I IFN suppression. Further focusing on the transcriptional control, motif enrichment analysis of down regulated genes in acLDL-loaded BMDMs showed a clear enrichment of genes harboring IRFs and IFN-sensitive response element (ISRE) motifs in the promoter regions (Figure 3D). These data suggest that the type-I IFN suppression induced by lipid loading, is likely mediated *via* suppression of the upstream IRFs.

### Foamy Peritoneal Macrophages Show a Similar Reduction in ISG Expression

Next, to investigate whether lipid loading affects the macrophage IFN response *in vivo* as well, we analyzed microarray data of foamy macrophages from mice in published datasets (GSE42061) (44). Macrophage foam cell formation increased the expression of *Abca1* and *Abcg1* in peritoneal macrophages from *Apoe*^−/−^ compared to WT mice (Figure 3E). In line with our *in vitro* data, we observed suppression of ISGs in foam cells from *Apoe*^−/−^ mice (Figure 3E). Pathway analysis of the down-regulated genes showed suppressed IFN response in the peritoneal macrophages (PMs) derived from hypercholesterolemic mice (Figure 3F). Upstream regulator analysis (Figure 3G) and motif (Figure 3H) analysis indicated lipid-suppressed IFN-signaling *via* IRFs in macrophages. Furthermore, the regulatory network of PMs derived from hypercholesterolemia mice (Supplementary Figure 3C) enclosed IRFs, including IRF3 and IRF7, that were highly connected to IFN-β and the affected biological processes. Taken together, our analyses revealed that cholesterol accumulation in macrophages dampens the IFN response, both *in vitro* and *in vivo*, which is likely through suppressing IRF expression and the subsequent type-I IFN production.

### Cholesterol-Loading in Human Macrophages Suppresses Type-I IFN Response

**Figure 4:**
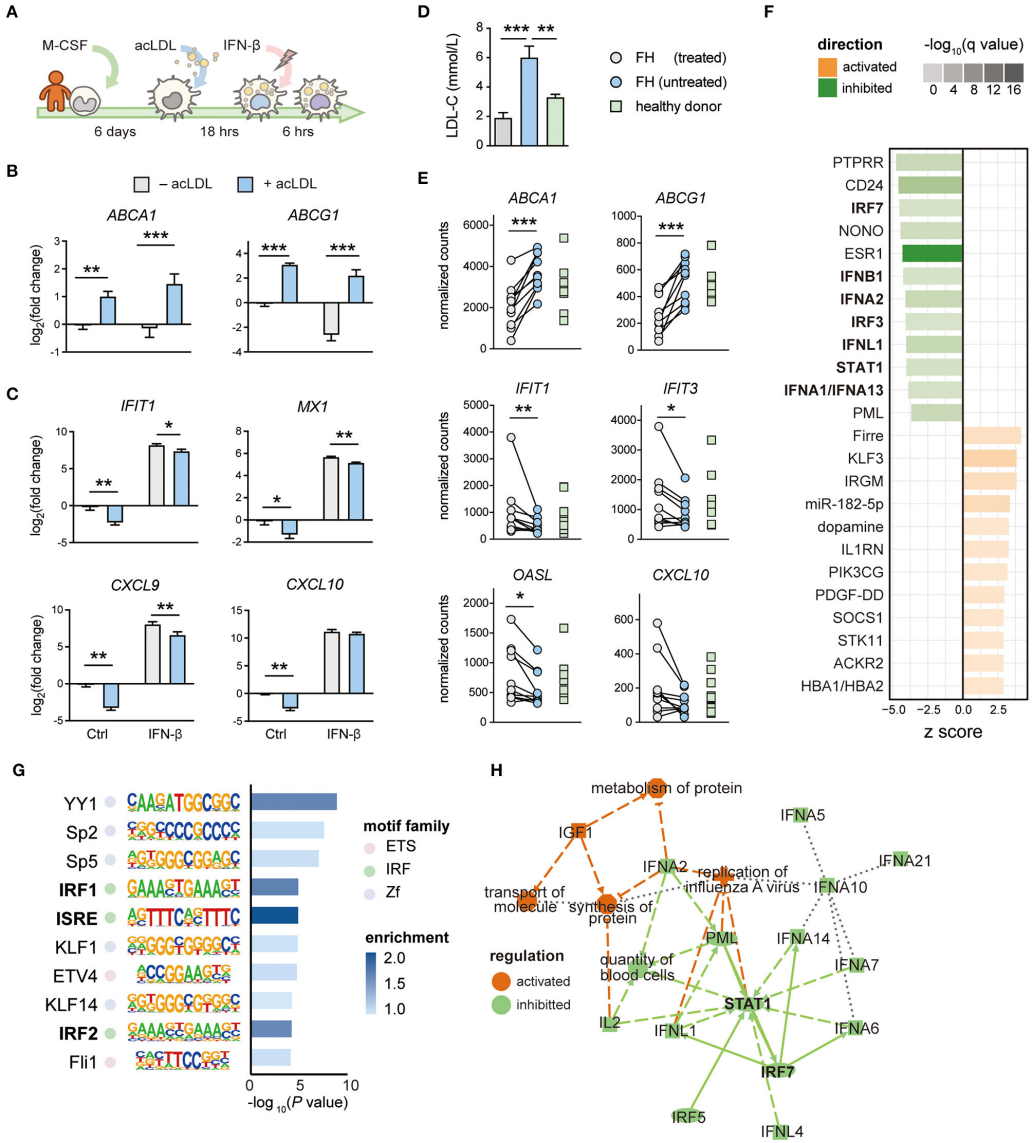
The IFN response is perturbated by lipid accumulation in human macrophages and monocytes from hypercholesterolemia patients. **(A)** Protocol of generating human monocyte-derived (foamy) macrophages (hMDM). Monocytes were differentiated with M-CSF for 6 days followed by 16-h (50 μg/mL) acLDL exposure. Next, the generated foamy hMDM were treated with (50 ng/mL) or without IFN-β for 6 h. **(B)** Cholesterol efflux transporter *ABCA1* and *ABCG1* were transcriptionally up-regulated by acLDL loading in foamy hMDM. **(C)** Interferon stimulated genes (*IFIT1, MX1, CXCL9*, and *CXCL10*) were transcriptionally downregulated in acLDL-loaded foamy hMDM **(B,C)** *n* = 5 biological replicates, data are represented as mean ± SEM, ^*^FDR < 0.05, ^**^FDR < 0.01, ^***^FDR < 0.001). **(D)** serum LDL-cholesterol (LDL-C) levels of treated/untreated familial hypercholesterolemia (FH) patients (*n* = 10) and healthy donors (HD, *n* = 9). **(E)** Gene expression of interferon stimulated genes (*IFIT1, IFIT3, OASL*, and *CXCL10*) from monocytes derived from treated/untreated FH patients and healthy donors. **(F)** Ingenuity Pathway Analysis revealed inhibition of interferon-related upstream regulators, such as IFNs, IRFs, and STAT1, in monocytes derived from FH patients. **(G)** Motif analysis showed an enrichment of the interferon-sensitive response element (ISRE) motif in the promoter region among the downregulated genes in FH monocytes. **(H)** Regulatory network analysis of differentially regulated genes showed an inhibition of highly interconnected IFN signatures. **(F–H)** raw data obtained from GSE6054.

To translate our findings to human, we applied the same lipid loading strategy using acLDL in hMDMs followed by IFN-β treatment (Figure 4A). Cholesterol loading was associated with an expected increased expression of the cholesterol efflux transporters *ABCA1* and *ABCG1* (Figure 4B). As we observed in mouse macrophages, cholesterol loading in hMDMs caused a reduced expression of ISGs, including *IFIT1, MX1, CXCL9*, and *CXCL10* (Figure 4C). Genes upstream of ISGs, including *IFNB1* (Supplementary Figure 4A) and IFN regulatory factors *IRF3, IRF7*, and *IRF8* (Supplementary Figure 4B) were also suppressed, supporting the concept of perturbated IFN-autocrine loop by cholesterol accumulation in human macrophages. In line with the mouse data, we did not observe this effect in non-ISG inflammatory genes such as *IL1B, IL6, CXCL8*, and *TNF*, confirming an IFN signaling-specific effect (Supplementary Figure 4C). This indicates that cholesterol loading also hampers the type-I IFN responses in human macrophages.

### Lipid Accumulation in Monocytes of Hypercholesterolemia Patients Results in Type-I IFN Suppression Which Is Reversed After Lipid-Lowering Treatment

We have previously shown that peripheral monocytes from FH patients accumulate lipids (55). To determine whether the suppressed IFN signature is also observed in monocytes of FH patients, we performed RNA-seq of peripheral monocytes derived from FH patients before and after lipid-lowering treatment by ezetimibe, statins, and/or PCSK9 antibodies, as well as age and gender-matched healthy donors (Supplementary Tables 1, 2). Indeed, serum LDL-C levels in untreated FH patients were significantly higher than samples obtained after treatment and from healthy donors (Figure 4D). RNA-seq analysis confirmed an elevated expression of *ABCA1* and *ABCG1*, whereas ISGs including *IFIT1, IFIT3, OASL*, and *CXCL10* were suppressed in untreated FH monocytes compared to monocytes from healthy donors (FDR < 0.05, Figure 4E). Interestingly, the suppressed gene expression of ISGs was restored after lipid-lowering treatment. To confirm these findings in monocytes from another FH patient cohort, we analyzed a publicly available dataset containing expression data of monocytes from FH patients and healthy donors (GSE60514) (45). Differential gene expression analysis showed that many ISGs were down regulated in monocytes derived from FH patients compared to healthy donors (Supplementary Figure 4D). Upstream regulator analysis on the differentially regulated genes confirmed inhibition of type-I IFNs, IRFs, and STAT1 (Figure 4F). In line with this, motif enrichment analysis identified ISRE as the most enriched promoter motif among the down regulated genes in FH patients (Figure 4G). Regulatory network analysis of the differentially expressed genes in monocytes from FH patients revealed STAT1 and IRF7 to be the central modulators of this network (Figure 4H). Thus, as observed in macrophages (mouse BMDMs, PMs and hMDMs), lipid accumulation in monocytes of FH patients also results in a deactivated type-I IFN response which can be restored by lipid-lowering therapy.

## Discussion

In the current study, we demonstrate that the expression of IFN-β and ISGs are affected by lipid-loading. We show that cholesterol accumulation *in vitro* and *in vivo* suppresses the type-I IFN response in both monocytes and macrophages. This cholesterol loading-induced immunomodulation is also observed by LXR activation and specifically affecting ISGs, but not other pro-inflammatory genes. By applying exogenous IFN-β to macrophages, we showed that the cholesterol-induced decreased ISG expression can be largely restored. Analysis of transcriptional profiles of FH monocytes confirmed this phenotype which was restored by lipid-lowering treatment in FH patients. Moreover, these analyses implicated a profound role of IRFs in the down regulation of type-I IFNs and the subsequent responses.

It has become increasingly clear that foam cell formation suppresses macrophage proinflammatory activation. Studies have shown that foamy peritoneal macrophages are less activated by TLR-ligand stimulation as a result of the accumulation of the LXR ligand desmosterol and suppressed activation of the pentose phosphate pathway (56, 57). Moreover, experiments comparing foamy vs. non-foamy plaque macrophages show that foamy macrophages in atherosclerotic lesions lack clear inflammatory characteristics (11, 13) and have identified LXR as a key transcriptional regulator in these cells (11). Here we show that foam cell formation specifically suppresses ISGs in macrophages, resembling an LXR-activated phenotype. In line with our results, desmosterol depletion in macrophages of atherosclerotic lesions increased the expression of ISGs and promoted the progression of atherosclerosis (58). Type-I IFNs have been shown to have a role in the resolution of inflammation by stimulation of IL-10 production as well as optimal macrophage activation and pro-inflammatory responses (23, 48). Moreover, type-I IFNs are mediators of many different human inflammatory and immune disorders and have also been implicated in atherosclerosis (15, 59). Blockade of type-I IFN signaling in macrophages suppressed atherogenesis, while IFN-β treatment accelerated atherosclerosis through the induction of the chemokine CCL5 which leads to increased monocyte recruitment to plaques (34). Altogether, this suggests that the cholesterol-induced down regulation of type-I IFN pathways is an anti-inflammatory, athero-protective characteristic of foamy macrophages.

Our main finding is that foam cells have reduced *Ifnb1* expression and IFN-β secretion resulting in a suppression of IFN-β-dependent ISG expression. The latter could be overcome by supplying exogenous IFN-β and suggests that at basal conditions there is type I IFN production by *in vitro* macrophages. Although we could not measure the low concentrations of IFN-β secreted by unstimulated macrophages, the rescue of ISG expression by low concentrations of IFN-beta (lower than measurable in our ELISA) does suggest autocrine/paracrine effects of type I IFN. Some ISGs, like *Ccl5* and *Cxcl9*, however, could not be rescued by IFN-β supplementation, which suggests that some IFN targets are regulated in a different manner and may for example utilize different IRFs to regulate gene expression. Future research should investigate whether blocking basal IFN-β production prevents autocrine/paracrine signaling of IFN-β and also leads to suppression of IFN-β-dependent ISG expression.

It has been described that type-I IFNs, including IFN-β, trigger both pro- and anti-inflammatory gene programs (60, 61). We observed this dual characteristic also after acLDL and IFN-β exposure. More specifically, IFN-β treatment suppressed the expression of certain proinflammatory genes (*IL1B* and *CXCL8*), while simultaneously the expression of other proinflammatory genes (*IL6* and *TNF*) was induced. acLDL treatment inhibited the transcription of ISGs, but induced the transcription of *IL1B* and *CXCL8*. This nicely confirms that inflammatory signaling pathways in macrophages can be differentially regulated through numerous interconnected modulatory processes. The cholesterol-mediated type-I IFN suppressive actions may contribute to the cholesterol-induced proinflammatory genes, which are suppressed by IFN, or *vice versa*.

IRFs are important immune orchestrators and not only trigger the transcription of ISGs upon IFN stimulation (23, 48, 62), but are also required for the production of type-I IFNs (63, 64) by recognizing the ISRE at these genes' promoter region (62, 65). IRF3 and IRF7 are highly homologous and are the key transcription factors for type-I IFN expression (63, 64, 66) directly binding to promoter regions (67) of genes encoding both IFN-α and IFN-β (63). In both mouse and human macrophages, cholesterol loading decreased the expression of *IRF3* and *IRF7* suggesting a central role in the suppressed IFN-β production. Interestingly, Chen et al. showed a negative feedback loop between LXR and IRF3 that is activated through LXR stimulation by oxLDL loading or GW3965 treatment of macrophages (68). Furthermore, it has been described that LXR can interact with STAT1 preventing STAT1 to bind to ISGs (69). Taken together, our results suggest a crosstalk between IFNs and cholesterol metabolism forming a feedback loop which might be mediated *via* IRF3 and/or IRF7.

The crosstalk between lipid metabolism and the IFN response could also contribute to the pathogenesis of infections. In the recent pandemic of coronavirus disease 2019 (COVID-19) caused by severe acute respiratory syndrome coronavirus 2 (SARS-CoV-2), disease morbidity and mortality are linked to reduced type-I IFN activities (70–72). Interestingly, *ex vivo* SARS-CoV-2 exposure of peripheral monocytes derived from healthy donors resulted in lipid droplet accumulation (73). Although data is lacking whether FH patients have an increased risk for severe COVID19, a meta-analysis suggested the potential favorable effect of lipid-lowering therapy (e.g., statins) on disease outcome (74). Other studies have indicated that PCSK9 inhibits *IFNB1* expression, and contributes to dampened antiviral cellular responses in Dengue fever patients, which could be abrogated by a PCSK9 inhibitor (75, 76). Because of the IFN enhancing effects of the PCSK9 inhibitor, the PCSK9 inhibitor was proposed as potential therapeutic for the treatment of COVID-19 (77, 78). Our results are in line with this hypothesis and show that lipid-lowering treatment in FH patients rescues the dampened IFN-responses in circulating monocytes. Targeting lipid-metabolism in monocytes using lipid-lowering treatment might thus be beneficial to promote anti-viral defense.

Future studies should investigate the mechanistic link between cholesterol exposure and the subsequent immune response modulations, including the type-I IFN response, in order to integrate these findings in the development of new therapeutic approaches for the treatment of e.g., cardiovascular and infectious disease.

## Data Availability Statement

The datasets presented in this study can be found in online repositories. The names of the repository/repositories and accession number(s) can be found below: RNA-seq data of the BMDMs treated with the LXR-agonist GW3965 or DMSO are deposited in the Gene Expression Omnibus (GEO) under the accession number: GSE193118. RNA-seq data of the monocytes from familial hypercholesterolemia patients and healthy subjects are deposited in GEO under the accession number: GSE192709 (processed data) and EGA (raw data).

## Ethics Statement

The studies involving human participants were reviewed and approved by the Amsterdam UMC and was conducted according to the principles of the Declaration of Helsinki. The patients/participants provided their written informed consent to participate in this study. The animal study was reviewed and approved by the Committee for Animal Welfare (University of Amsterdam).

## Author Contributions

LW, H-JC, MH, and MW designed the research or gave critical input to the design. LW and H-JC performed the majority of experiments with contributions from CR and AN (BMDM experiments), GG (human macrophage experiments), RS (IFN-β ELISA), JK (FH monocytes). LW and H-JC analysed the data and generated graphical representation. LW and H-JC wrote the manuscript and JK, MH, and MW gave critical feedback. MH and MW supervised the study. All authors contributed to the article and approved the submitted version.

## Funding

This work was supported by the European Union (EU) Horizon 2020 program EPIMAC (SEP-210163258). AN was supported by Amsterdam Cardiovascular Sciences (ACS) and the Netherlands Heart Foundation (Dekker grant 2020T029). JK received a VENI grant from ZonMW (91619098) and a Senior Scientist Dekker grant from the Netherlands Heart Foundation (03-004-2021T045). MH was supported by a Marie Sklodowska Curie Action individual fellowship (MSCA-IF-EF 895411). MW was supported by Amsterdam UMC, Amsterdam Cardiovascular Sciences, the Netherlands Heart Foundation (CVON GENIUS and GENIUSII 2017-20), Spark-Holding BV (2015B002 and 2019B016), Fondation Leducq (Transatlantic Network Grant No. 16CVD01), and ZonMW (open competition 09120011910025).

## Conflict of Interest

The authors declare that the research was conducted in the absence of any commercial or financial relationships that could be construed as a potential conflict of interest.

## Publisher's Note

All claims expressed in this article are solely those of the authors and do not necessarily represent those of their affiliated organizations, or those of the publisher, the editors and the reviewers. Any product that may be evaluated in this article, or claim that may be made by its manufacturer, is not guaranteed or endorsed by the publisher.

## Supplementary Material

Supplementary Figure 1 acLDL exposure suppresses interferon-responsive genes and transcription factors in BMDMs. mRNA expression of **(A)** *Cxcl9* and *Ccl5* and **(B)** *Stat1, Stat2*, and *Irf7* in unstimulated, acLDL and/or IFN-β (50 ng/mL) stimulated BMDMs measured by qPCR. ^*^*P* < 0.05, ^**^*P* < 0.01, ^***^*P* < 0.001. **(A,B)** *n* = 3 biological replicates per group.

Supplementary Figure 2 Transcriptional analysis on BMDMs stimulated with different concentration of IFN-β **(A)** mRNA expression of the cholesterol efflux transporters *Abca1* and *Abcg1* in BMDMs after a total of 24 h of acLDL exposure combined with different concentrations (1.5 pg/mL to 50 ng/mL) of IFN-β for 6 h of stimulation. **(B)** the transcriptional inhibition of *Cxcl9* and *Ccl5* that was induced by acLDL loading was unaffected after IFN-β exposure. **(C)** mRNA expression of pro-inflammatory cytokine *Il6* was unaffected by acLDL but induced by IFN-β in a dose-dependent manner. **(A–C)** *n* = 3 biological replicates per group.

Supplementary Figure 3 Transcriptomic analysis identifies IRFs as important regulators of the suppressed IFN response in foamy macrophages *in vitro* and *in vivo*. **(A)** Pathway enrichment analysis of significantly upregulated genes (FDR < 0.05) and **(B)** IPA regulatory network analysis of transcriptional profile in acLDL-loaded macrophages. **(C)** IPA regulatory network analysis of the transcriptional profile of foamy PMs. **(A,B)** GSE118656. **(C)** GSE42061.

Supplementary Figure 4 The transcription of IFN-independent pro-inflammatory cytokines and chemokines was induced or unaffected by lipid-loading in human macrophages. **(A)** Type-I Interferon (*IFNB1*) and **(B)** interferon regulatory factors (*IRF3, IRF7*, and *IRF8*) were transcriptionally downregulated in acLDL-loaded foamy hMDM. **(C)** Gene expression of the non-interferon stimulated genes, *IL1B* and *CXCL8*, was induced in human monocyte-derived macrophages upon lipid loading, but suppressed by IFN-β treatment. Gene expression of *IL6* and *TNF* remained unaltered after acLDL loading (*n* = 5 biological replicates, data are represented as mean ± SEM. ^**^FDR0.01, ^***^FDR < 0.001). **(D)** Volcano plot depicting up- (orange) and down- (green) regulated genes of monocytes derived from familial hypercholesterolemia patients compared to these of healthy donors.

Supplementary Table 1 Baseline characteristics FH patients and healthy controls.

Supplementary Table 2 LDL-C lowering effect per individual FH patient.
