# Supplementary figures and images for: Monocyte and Macrophage Lipid Accumulation Results in Down-Regulated Type-I Interferon Responses
Source: Front Cardiovasc Med. 2022 Feb 10;9:829877. doi: 10.3389/fcvm.2022.829877 (PMC8869252; doi:10.3389/fcvm.2022.829877)

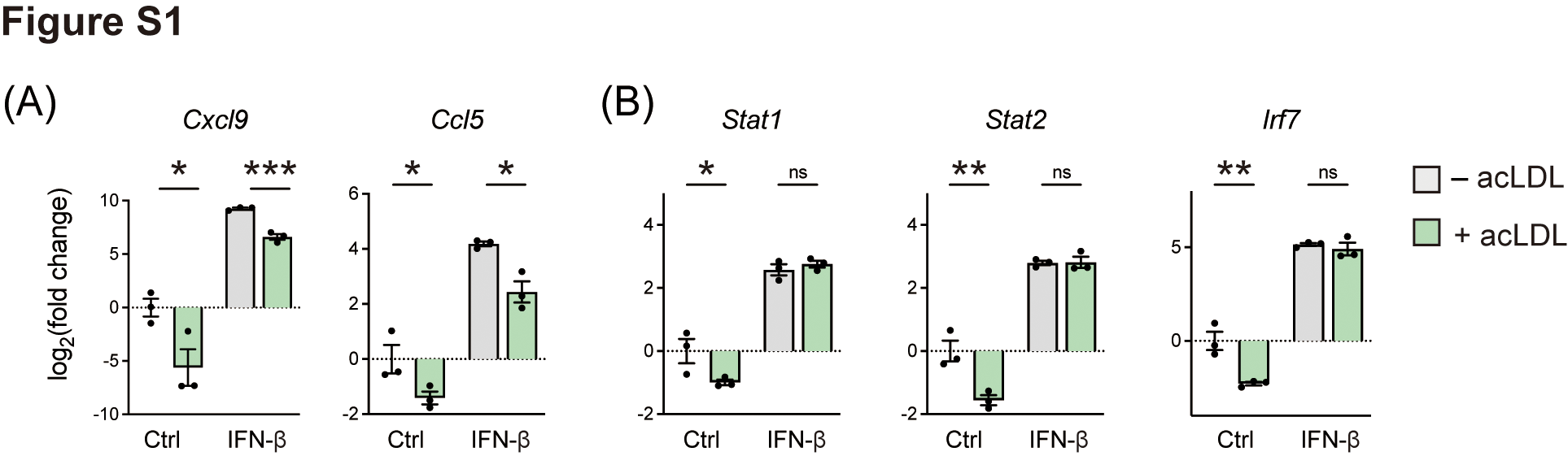

Supplement: Supplementary Figure 1 — acLDL exposure suppresses interferon-responsive genes and transcription factors in BMDMs. mRNA expression of (A) Cxcl9 and Ccl5 and (B) Stat1, Stat2, and Irf7 in unstimulated, acLDL and/or IFN-β (50 ng/mL) stimulated BMDMs measured by qPCR. *P < 0.05, **P < 0.01, ***P < 0.001. (A,B) n = 3 biological replicates per group. [file Image_1.TIF]

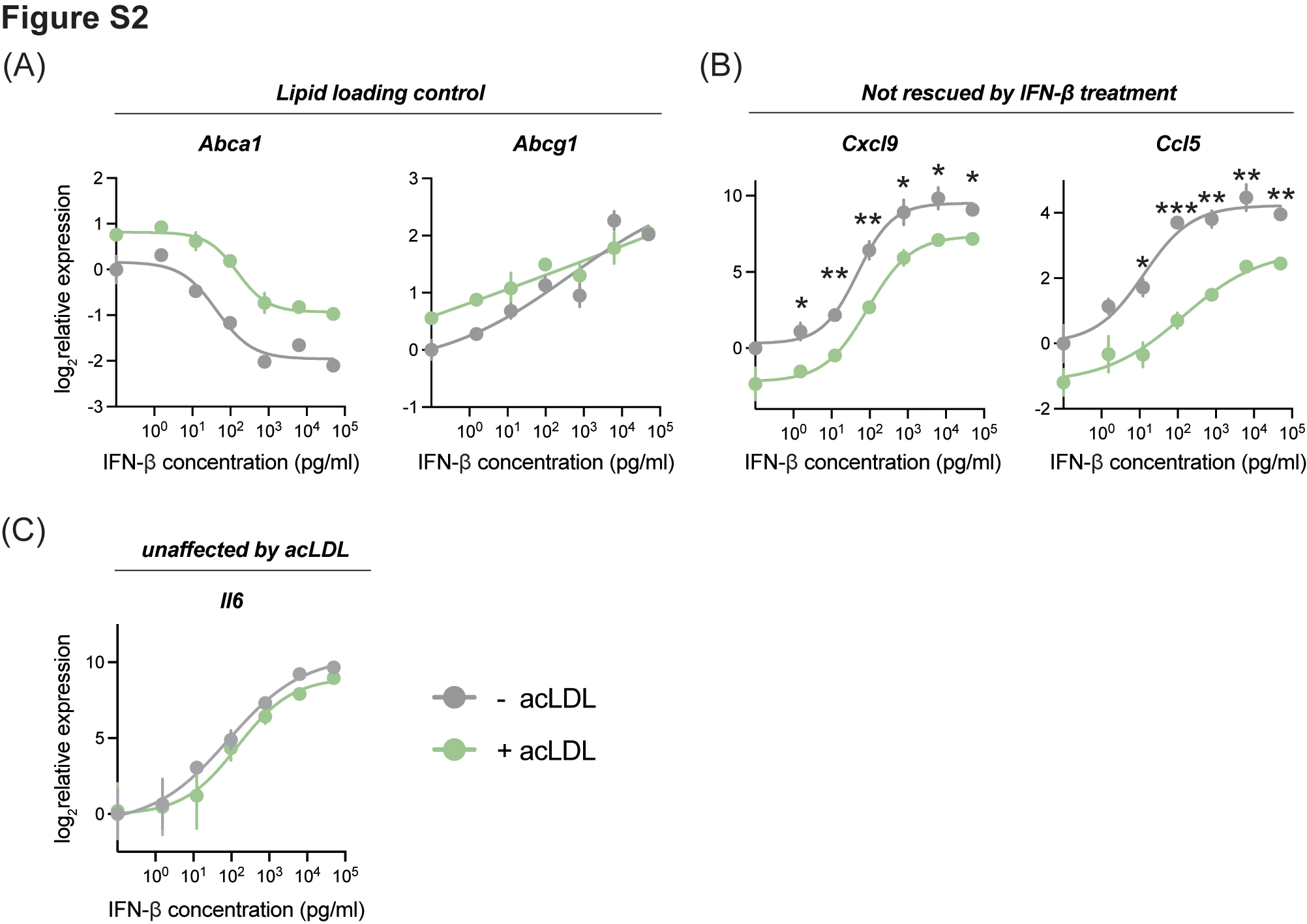

Supplement: Supplementary Figure 2 — Transcriptional analysis on BMDMs stimulated with different concentration of IFN-β (A) mRNA expression of the cholesterol efflux transporters Abca1 and Abcg1 in BMDMs after a total of 24 h of acLDL exposure combined with different concentrations (1.5 pg/mL to 50 ng/mL) of IFN-β for 6 h of stimulation. (B) the transcriptional inhibition of Cxcl9 and Ccl5 that was induced by acLDL loading was unaffected after IFN-β exposure. (C) mRNA expression of pro-inflammatory cytokine Il6 was unaffected by acLDL but induced by IFN-β in a dose-dependent manner. (A–C) n = 3 biological replicates per group. [file Image_2.TIF]

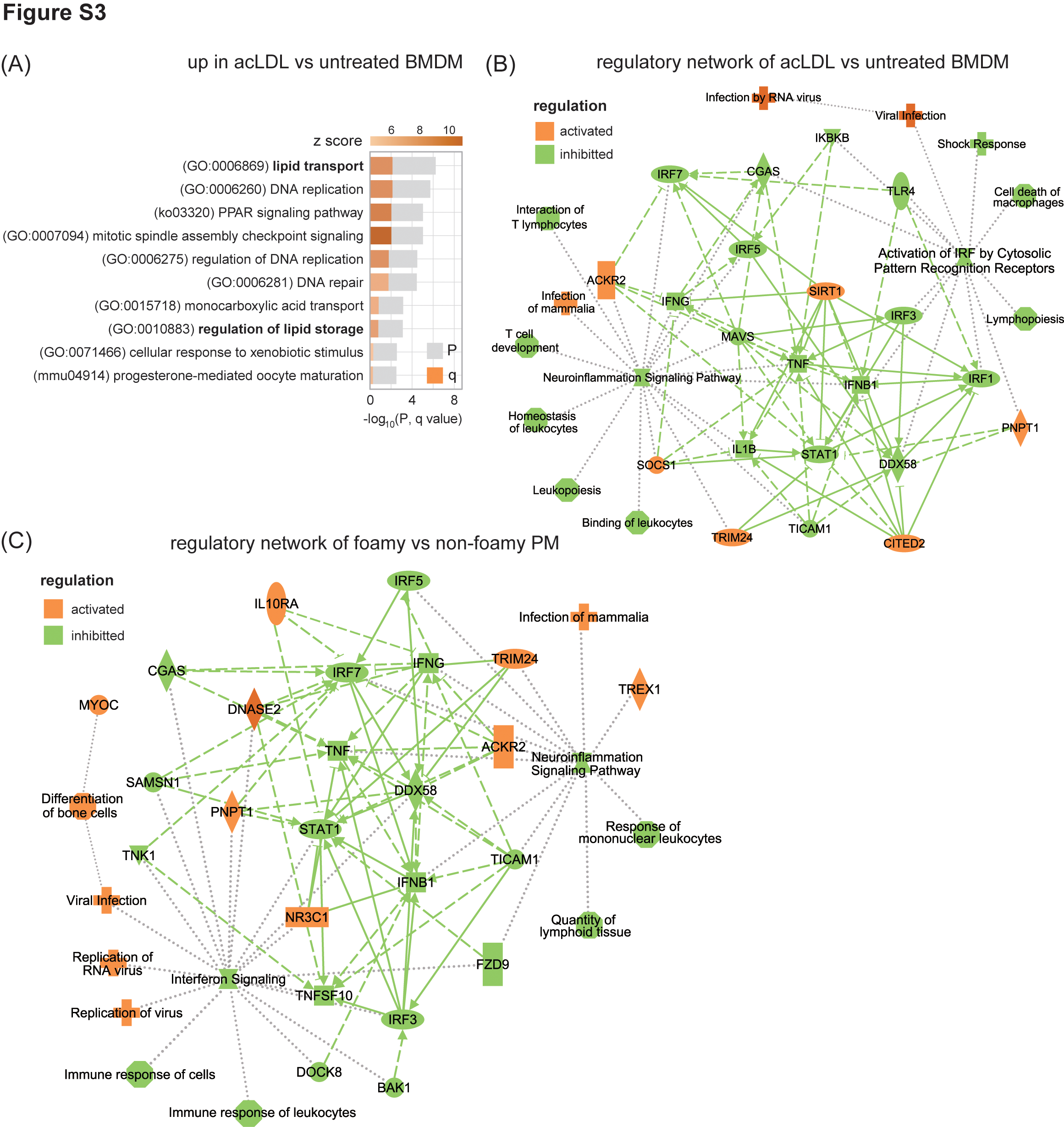

Supplement: Supplementary Figure 3 — Transcriptomic analysis identifies IRFs as important regulators of the suppressed IFN response in foamy macrophages in vitro and in vivo. (A) Pathway enrichment analysis of significantly upregulated genes (FDR < 0.05) and (B) IPA regulatory network analysis of transcriptional profile in acLDL-loaded macrophages. (C) IPA regulatory network analysis of the transcriptional profile of foamy PMs. (A,B) GSE118656. (C) GSE42061. [file Image_3.TIF]

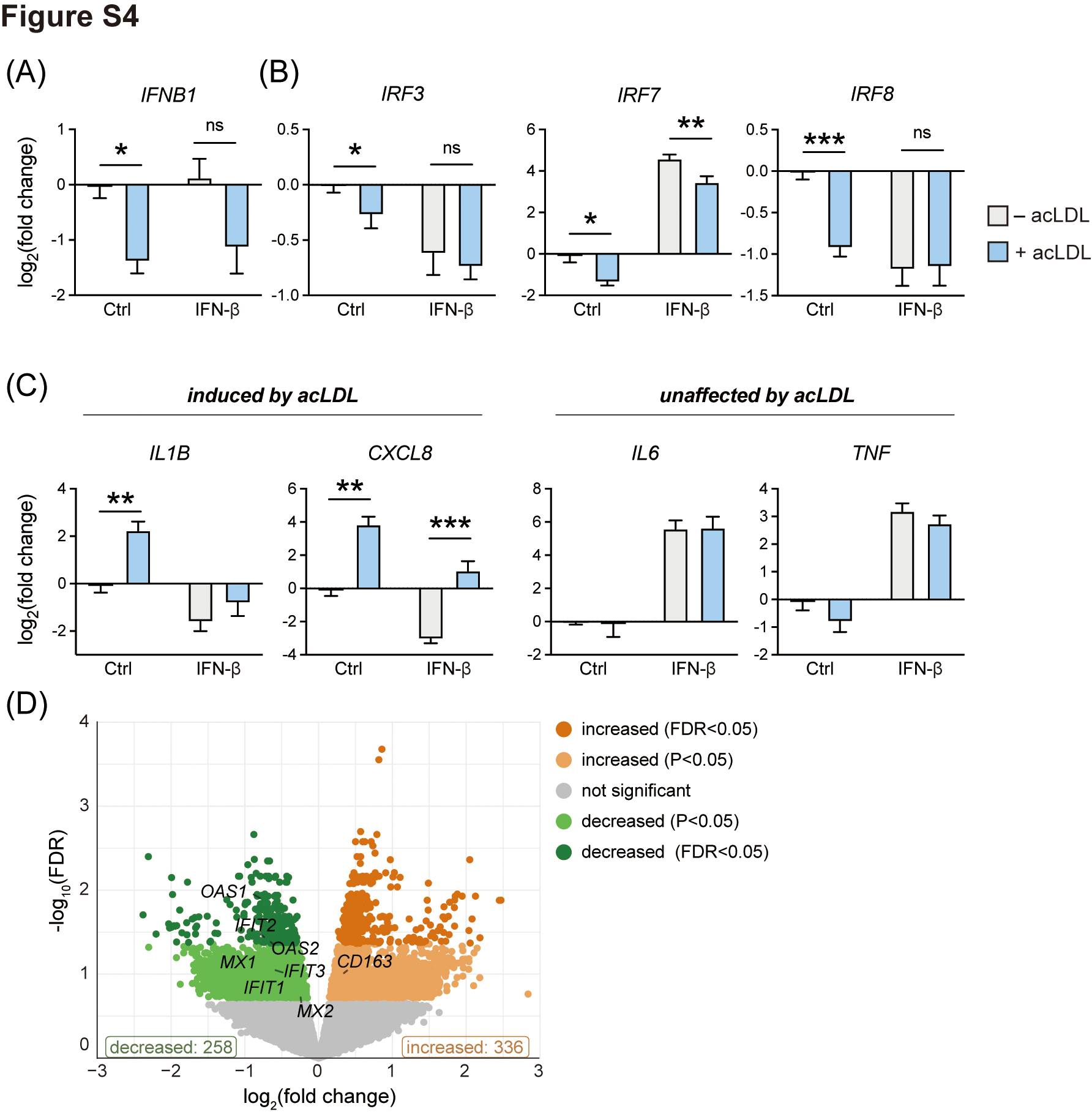

Supplement: Supplementary Figure 4 — The transcription of IFN-independent pro-inflammatory cytokines and chemokines was induced or unaffected by lipid-loading in human macrophages. (A) Type-I Interferon (IFNB1) and (B) interferon regulatory factors (IRF3, IRF7, and IRF8) were transcriptionally downregulated in acLDL-loaded foamy hMDM. (C) Gene expression of the non-interferon stimulated genes, IL1B and CXCL8, was induced in human monocyte-derived macrophages upon lipid loading, but suppressed by IFN-β treatment. Gene expression of IL6 and TNF remained unaltered after acLDL loading (n = 5 biological replicates, data are represented as mean ± SEM. **FDR0.01, ***FDR < 0.001). (D) Volcano plot depicting up- (orange) and down- (green) regulated genes of monocytes derived from familial hypercholesterolemia patients compared to these of healthy donors. [file Image_4.TIF]
