# Supplementary material for: Monocyte and Macrophage Lipid Accumulation Results in Down-Regulated Type-I Interferon Responses
Source: Front Cardiovasc Med. 2022 Feb 10;9:829877. doi: 10.3389/fcvm.2022.829877 (PMC8869252; doi:10.3389/fcvm.2022.829877)
Supplement: Supplementary Table 1 — Baseline characteristics FH patients and healthy controls. [file Table_1.docx]

**Table S1. Baseline characteristics FH patients and healthy controls**

|  | **FH patients**  (n=10) | **Healthy controls**  (n=9) | **P value** |
| --- | --- | --- | --- |
| Age, years | 42.7 (10.2) | 41.2 (11.7) | 0.772 |
| Sex, n male (%) | 8 (72) | 7 (78) | 0.906 |
| BMI, kg/m^2^ | 25.2 (1.9) | 24.3 (2.5) | 0.403 |
| Smoking, n never/past (%) | 8/2 (80/20) | 6/3 (67/33) | 0.156 |
| SBP, mmHg | 120 (11) | 123 (5) | 0.470 |
| DBP, mmHg | 80 (8) | 82 (6) | 0.563 |
| hs-CRP, mg/L, median [IQR] | 0.9 [0.6-1.6] | 0.5 [0.3-0.6] | **0.022** |
| Total cholesterol, mmol/L^*^ | 8.1 (2.7) | 5.2 (0.7) | **0.009** |
| LDL-cholesterol, mmol/L^*^ | 6.0 (2.5) | 3.3 (0.6) | **0.007** |
| HDL-cholesterol, mmol/L^*^ | 1.3 (0.4) | 1.5 (0.3) | 0.253 |
| Triglycerides, mmol/L†, median [IQR] | 1.34 [0.77-2.74] | 0.94 [0.80-1.18] | 0.243 |
| ApoB, g/L, median [IQR] | 1.52 [1.24-1.97] | 0.80 [0.75-1.02] | **<0.001** |
| Leukocytes, 10^9^/L | 5.7 (1.6) | 5.3 (0.6) | 0.552 |
| Neutrophils, 10^9^/L | 3.2 (1.7) | 2.8 (0.4) | 0.534 |
| Lymphocytes, 10^9^/L | 1.8 (0.4) | 1.8 (0.2) | 0.876 |
| Monocytes, 10^9^/L | 0.46 (0.07) | 0.48 (0.12) | 0.721 |
| Hemoglobin, mmol/L | 9.4 (0.6) | 9.0 (0.7) | 0.179 |
| Thrombocytes, 10^9^/L | 246 (62) | 267 (51) | 0.453 |
| Glucose, mmol/L | 5.2 (0.5) | 5.2 (0.4) | 0.812 |

Data are mean (SD), median [interquartile range], or n (%). ApoB, apolipoprotein B; BMI, body mass index; DBP, diastolic blood pressure; HDL, high-density lipoprotein; hs-CRP, high-sensitivity C-reactive protein; LDL, low-density lipoprotein; SBP, systolic blood pressure
^*^ To convert to mg/dL, multiply by 38.7; † To convert to mg/dL, multiply by 88.6
