# Supplementary material for: Monocyte and Macrophage Lipid Accumulation Results in Down-Regulated Type-I Interferon Responses
Source: Front Cardiovasc Med. 2022 Feb 10;9:829877. doi: 10.3389/fcvm.2022.829877 (PMC8869252; doi:10.3389/fcvm.2022.829877)
Supplement: Supplementary Table 2 — LDL-C lowering effect per individual FH patient. [file Table_2.docx]

**Table S2. LDL-C lowering effect per individual FH patient**

| **subject** | **sex** | **age** | **FH type** | **BMI** | **treatment** | **ΔLDL-C,  mmol/L^*^** | **ΔLDL-C,  %** | **post-treatment LDL-C, mmol/L^*^** |
| --- | --- | --- | --- | --- | --- | --- | --- | --- |
| **I** | F | 35 | LDLR | 24.4 | P, E | -1.93 | -45 | 2.35 |
| **II** | M | 24 | Clin | 23.0 | S | -2.67 | -66 | 1.40 |
| **III** | M | 47 | Clin | 25.5 | P | -4.24 | -96 | 0.19 |
| **IV** | M | 56 | LDLR | 27.4 | P, E | -5.31 | -57 | 4.05 |
| **V** | M | 39 | LDLR | 23.2 | S, P, E | -9.72 | -87 | 1.42 |
| **VI** | M | 38 | LDLR | 23.0 | S, E | -3.84 | -56 | 2.98 |
| **VII** | M | 52 | APOB | 25.2 | S, E | -0.94 | -24 | 2.91 |
| **VIII** | M | 43 | Clin | 25.1 | S, E | -3.64 | -70 | 1.57 |
| **IX** | F | 56 | Clin | 28.7 | P, E | -4.97 | -85 | 0.88 |
| **X** | M | 37 | Clin | 26.0 | P, E | -3.57 | -76 | 1.10 |
|  |  |  |  | **mean (SD)** | | **-4.08 (2.38)** | **-66 (22)** | **1.89 (1.16)** |

APOB indicates mutation in apolipoprotein B gene; Clin, clinical diagnosis of familial hypercholesterolemia (i.e., no FH mutation was found by genetic testing); E, ezetimibe; F, female; LDL-C, low density lipoprotein cholesterol; LDLR, mutation in low density lipoprotein receptor gene; M, male; P, proprotein convertase subtilisin/kexin type 9 antibody; S, statin

* To convert to mg/dL, multiply by 38.7
